# Synthetic repurposing of drugs against hypertension: a datamining method based on association rules and a novel discrete algorithm

**DOI:** 10.1186/s12859-020-03644-w

**Published:** 2020-07-16

**Authors:** Yosef Masoudi-Sobhanzadeh, Ali Masoudi-Nejad

**Affiliations:** Laboratory of Systems Biology and Bioinformatics (LBB), Institute of Biochemistry and Biophysics, University of Tehran, Tehran, Iran

**Keywords:** Data mining, Drug repurposing, Hypertension, Optimization algorithm, Synthetic repurposing

## Abstract

**Background:**

Drug repurposing aims to detect the new therapeutic benefits of the existing drugs and reduce the spent time and cost of the drug development projects. The synthetic repurposing of drugs may prove to be more useful than the single repurposing in terms of reducing toxicity and enhancing efficacy. However, the researchers have not given it serious consideration. To address the issue, a novel datamining method is introduced and applied to repositioning of drugs for hypertension (HT) which is a serious medical condition and needs some improved treatment plans to help treat it.

**Results:**

A novel two-step data mining method, which is based on the If-Then association rules as well as a novel discrete optimization algorithm, was introduced and applied to the synthetic repurposing of drugs for HT. The required data were also extracted from DrugBank, KEGG, and DrugR+ databases. The findings indicated that based on the different statistical criteria, the proposed method outperformed the other state-of-the-art approaches. In contrast to the previously proposed methods which had failed to discover a list on some datasets, our method could find a combination list for all of them.

**Conclusion:**

Since the proposed synthetic method uses medications in small dosages, it might revive some failed drug development projects and put forward a suitable plan for treating different diseases such as COVID-19 and HT. It is also worth noting that applying efficient computational methods helps to produce better results.

## Background

Hypertension (HT) is a long-term medical condition, in which blood circulates anomalously through the vessels. In terms of the nature of HT, the patients are divided into two groups, which are as follows [1]:

i) The Primary HT: some genetic factors as well as unhealthy lifestyles such as having a salty diet, smoking, drinking alcohol, stresses and strains, overweight etc. [2] all have an important role in inducing HT. More than 90% of the hypertensive patients who are mostly adults, are placed in this category.
ii) The Secondary HT: some medical conditions such as the chronic kidney illnesses might also give rise to HT [3]. Less than 10% of the patients, whose HT may be reduced through treating the main condition [4], are placed in this group.

According to the world health organization, about 1.3 billion people around the world, which is a remarkable number, suffer from HT [5]. Most of these patients who are from the low and middle-paid countries [6], need some proper therapeutic plans such as drug repurposing methods which could yield the desired effect [7]. Drug repurposing or drug repositioning process, which may be the best option to treat diseases, brings about some big advantages as follows: First, it may make the finding treatment plan against HT a cost-effective and time-saving process. Second, it might be useful in treating both the orphan and rare HT diseases such as COVID-19, considering the fact that drug companies cannot afford to develop new molecular entities or cannot develop a suitable medication in a reasonable time order. Third, it might prove that the existing drugs have many hidden medical benefits in treating HT, in view of the fact that such advantages have not been detected before.

Beside taking advantage of drug repurposing, The synthetic repurposing of Drugs, in which instead of recommending a specific drug, a combination of two or more medicines is prescribed, might yield various advantages as follows:

i) It might lessen the medicines’ toxicity through applying them in small doses [8], which in turn can lead to reviving the previous drug development projects which have failed due to facing some biological problems such as bioavailability or large amounts of toxicity.
ii) It may increase the effectualness of drugs and yield some better treatment methods. In contrast to a single therapy, a proper combination of drugs can produce much better synergic effect and have more control over the disease. However, the drug-drug adverse reactions are considered to be the main challenge in front of the researchers [9].
iii) It may pave the way for developing a new research branch of drug repurposing and expanding the drug usages in a wide range of diseases [10], which in turn can help pharmaceutical companies or pharmacologists determine the doses of drugs and address their technical issues.
iv) The results obtained from the synthetic repurposing of drugs may also be applied to a combination therapy, in which a drug can increase the effectualness of one particular drug when combined with it [11]. There is a marked difference between the synthetic treatment and combination therapy concepts. The first one is based on a synthesis of different drugs instead of a given medication, whereas the second one combines one or more medications with a given drug [12]. In other words, in the synthetic repurposing method, a combination of two drugs (A and B) is used instead of a given drug (C), whereas in the combination therapy, a combination of a given drug (C) and another drug (E) is being used.

The present study aims to introduce the synthetic repurposing of drugs as a useful approach to treat various diseases such as HT. For this purpose, a novel datamining method, which is based on our proposed algorithm and the association rules, is presented. The approach employed to conducting the research, consists of two main parts as follows: First, the If-Then association rules are applied to a large volume of data to extract information about the drug-target interactions, the drug-drug adverse reactions, and the drug-diseases data. Second, the discrete version of the proposed discrete algorithm (Trader) [13] is introduced and used to discover the synthetic lists which might be useful in controlling HT.

In the present study, the related works on the repurposing of drugs are examined and categorized from a computational perspective, which are listed as follows:

i) The machine learning-based researches: in these researches, the existing data are examined to discover the relationship between inputs and outputs [14]. Overall, until recently, three types of machine learning methods, including supervised, semi-supervised, and unsupervised have been applied to the scope of drug discovery processes [15]. It has been also shown that some modified and improved versions of the present approaches such as deep neural networks could yield better predictive models [16], and overfitting and insufficient amounts of data have been the main challenges in front of the researchers who have been engaged in generating an appropriate predictive model [17–19].
ii) The theory-based researches: normally, researchers formulate the relationship among the biological entities based on the numerical and experimental experiences [20, 21]. For this purpose, they have proposed many mathematical equations to calculate the drugs’ structural similarities [22, 23] and based on the similarity score, they have examined the role of the analogous drugs in treating diseases [24]. The fact that the drugs with similar structures (scores) might be replaced with one another, is of the utmost importance. However, it has been reported that the theory-based methods are not applicable to the most projects and to qualify, they need to meet some other criteria [25].
iii) The graph and network theory-based researches: these studies employ a graph or a network to show the communication among the biological components [26]. For this purpose, first the graph algorithms are applied to the generated network [27, 28], and then the hidden relations are detected [29]. Although these techniques are confronted with minimal validity challenges and can produce significant results, they are not applicable to the most drug repurposing cases because the biological elements act according to the hypergraph concepts [30, 31].
iv) The text mining-based researches: in these studies, different algorithms are applied to delve into a massive volume of raw data and gather the desired ones [32]. The prevalent drug repositioning strategies which are employed to apply this approach, are as follows: K-means, KNN, the association rules, and optimization algorithms [33, 34]. For best results, it is essential to organize the data effectively and apply the state-of-the-art algorithms. Our proposed method, which employs both the association rules (If-Then) [35] and the novel discrete algorithm (Trader), fits into this category of the related works.
v) The ensemble method-based researches: in such research projects, various techniques are combined in several different ways [36] to generate an efficient tool for predicting and discovering the hidden benefits of the drugs [37]. For example, some researchers have mixed different aspects of the computational methods to obtain a suitable predictive model [38–40]. However, from the biological perspective, it has been shown that the simple techniques are sometimes better than the complex ones [41], and the ensemble methods’ overfitting possibilities might be more than the other approaches’.

## Results

The newly proposed method for the synthetic repurposing of drugs was implemented in the MATLAB programming language and then compared with the four state-of-the-art algorithms, including the discrete symbiotic optimization search (DSOS) [42], the forest optimization algorithm (FOA) [43], the world competitive contests algorithm (WCC) [44], and the cuckoo optimization (CUK) algorithm [45]. Afterwards, the above-mentioned state-of-the-art algorithms were applied to the generated datasets which have been presented in Table 1. Next, some candidate medicines were selected from medications which have been suggested for use in controlling HT by the different proposed treatment methods. In the datasets, there exist three pieces of information about every drug, which are as follows: i) the total number of a drug’s targets, including the main targets and the side effects, ii) the total number of a drug’s targets which are effective in controlling hypertension, and (iii) the total number of the drugs which have a target in common with one of the main targets of the specified drug. For instance, in the case of Nicardipine which interacts with 15 targets, only 4 targets play a key role in treating hypertension, and the remaining 11 targets are regarded as just some side effects. There are also 40 drugs which have at least an interaction with the main targets of Nicardipine. The present study aims to substitute a specified drug such as Nicardipine with an optimal subset of drugs which help control hypertension.

**Table 1:** The properties of the generated datasets

DrugBank Id	Drug name	Abbr	Chemical structure	TNT	TNMT	TNC
DB00519	Trandolapril	TRA		1	1	16
DB00335	Atenolol	ATE		2	1	26
DB00521	Carteolol	CAR		2	2	28
DB00622	Nicardipine	NIC		15	4	40
DB01023	Felodipine	FEL		13	5	50
DB01115	Nifedipine	NIF		8	5	50
DB00401	Nisoldipine	NIS		5	5	50
DB00590	Doxazosin	DOX		6	3	112
DB00457	Prazosin	PRA		6	3	112

*TNT* The total number of targets, *TNMT* The total number of main targets, *TNC* The total number of candidates

**Fig. 1:**
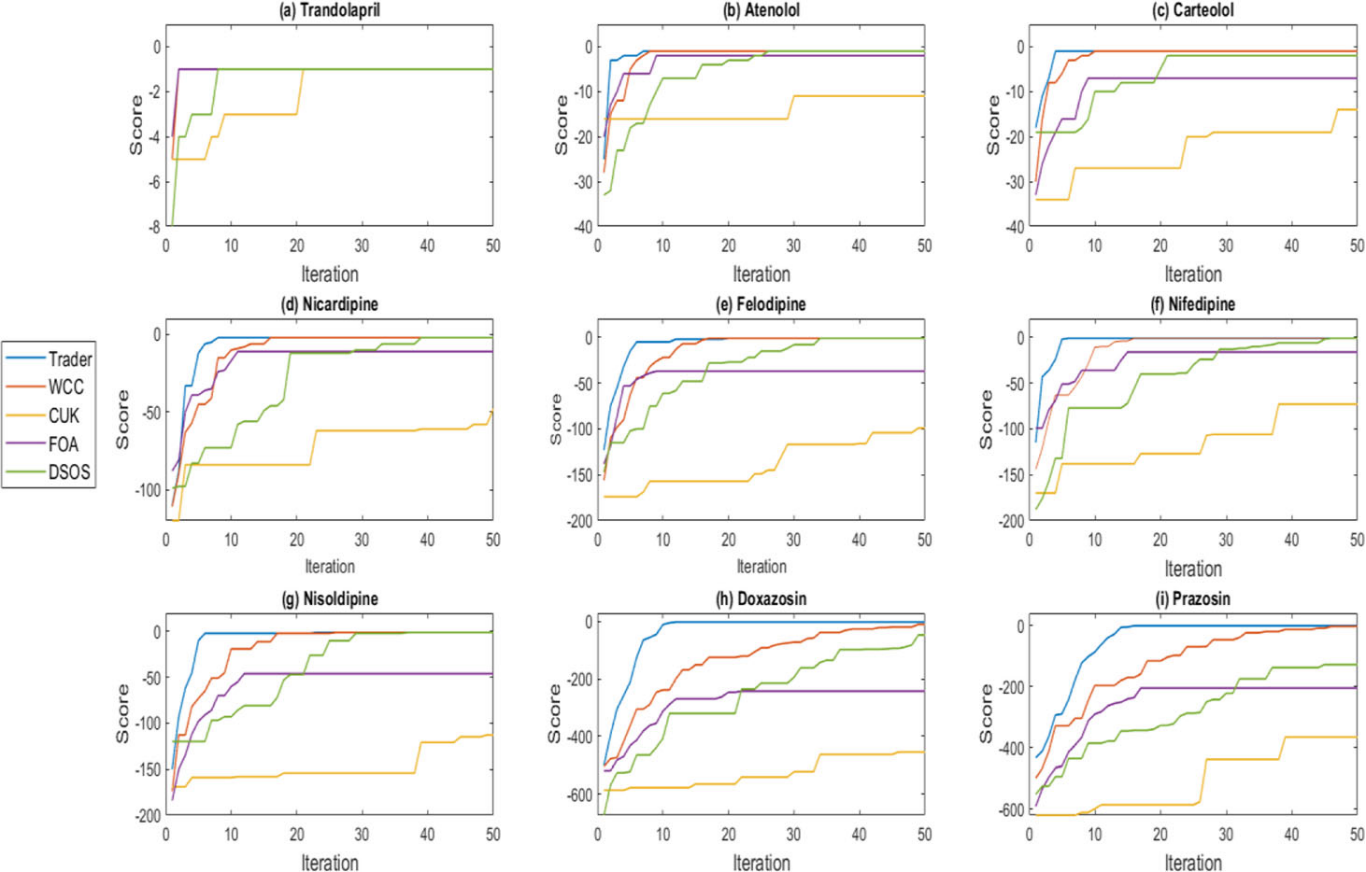
The convergence behavior of the algorithms on the generated datasets. The convergence of the algorithms on the **a** Trandolapril, **b** Atenolol, **c** Carteolol, **d** Nicardipine, **e** Felodipine, **f** Nifedipine, **g** Nisoldipine, **h** Doxazosin, and **i** Prazosin datasets. For the datasets with small sizes, the performance of the algorithms is almost the same. However, through increasing the total number of candidate drugs (the size of the problem), algorithms display different functionality. Trader, WCC, DSOS, FOA, and CUK, respectively are among the algorithms which are considered to have the proper convergence behavior

The results obtained from applying the algorithms to the datasets have been divided into two categories, which are as follows:

i) In the first class of evaluations, the performance of the algorithms was examined in terms of convergence, stability, and some statistical criteria such as the *P*-value, the standard deviation (STD). The stochastic operations of the optimization algorithms generate various results in their different runs. For this reason, each algorithm was individually executed 50 times, and then the generated data were analyzed. Each algorithm was executed under similar circumstances and invoked the same number of the score functions. Figure 1 represents the convergence of the algorithms on the generated datasets. The horizontal and vertical axes show the iteration number and the best-obtained score, respectively. When the size of the problem or the candidate drugs is small, most of the algorithms can choose the best possible subset of medicines to cure HT. However, both their performance and their convergence are reduced when the total number of the candidate drugs rises, which in turn can lead to their failure to acquire the best answer to the synthetic repurposing of drugs. For instance, Although the FOA algorithm has acquired the best solution to the Trandolapril dataset, it does not produce the best synthetic medicines on the remaining datasets and therefore falls into the local optima solutions.

**Fig. 2:**
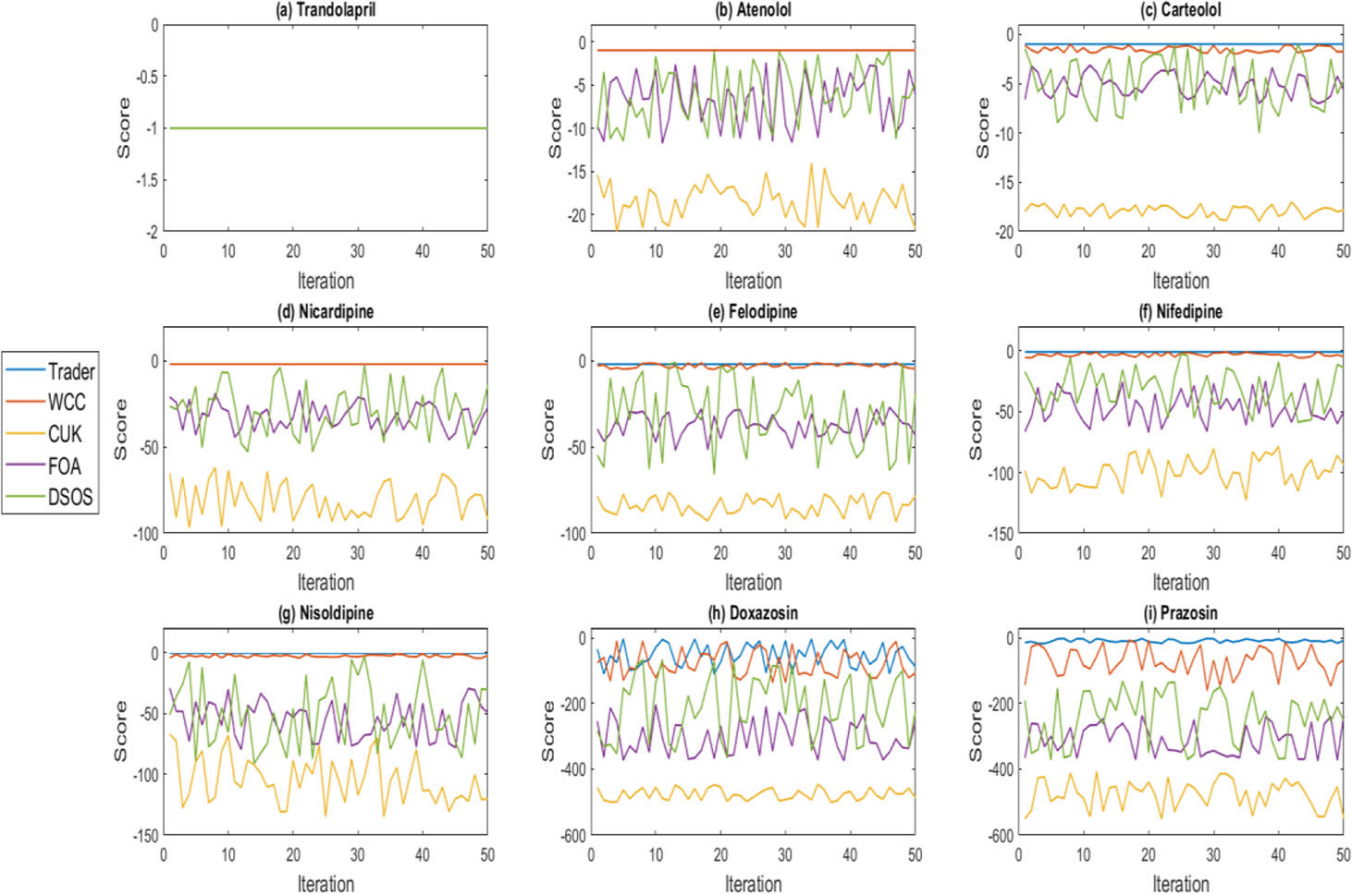
The stability behavior of the algorithms on the generated dataset. The stability of the algorithms on the **a** Trandolapril, **b** Atenolol, **c** Carteolol, **d** Nicardipine, **e** Felodipine, **f** Nifedipine, **g** Nisoldipine, **h** Doxazosin, and **i** Prazosin datasets. Trader is remarkably more stable than the others and delivers better results on the datasets except for Trandolapril and Nicardipine. Moreover, Trader’s performance is better than the others’ because the performance of the other algorithms lowers through increasing the total number of candidate drugs

The principles of the meta-heuristic algorithms are approximately the same. For instance, these algorithms generate some random potential answers to a problem and, then, try to improve them based on some random operations [46]. Therefore, they must be executed at least 30 times, and their performance should be evaluated based on the produced data [47]. An algorithm which can yield very similar results in disparate runs, is more stable than the others and consequently, its generated outcomes should be better than the others’. Figure 2 demonstrates the algorithms’ stability on the created datasets in 50 distinct executions.

In Fig. 2, the horizontal and vertical axes present the number of runs and the best-obtained score values, respectively. Since all of the algorithms produce the best possible results on Trandolapril in all of their executions, the generated results overlap. The stability of each algorithm will differ from that of the other ones as the number of the candidate drugs increases. Overall, Trader, WCC, DSOS, FOA, and CUK, respectively are the best algorithms in terms of the stability power.

Table 2, which shows the algorithms’ performance on the generated datasets in 50 distinct executions, has been provided to examine the functionality of the algorithms accurately. For different executions, this table presents the following information: (i) the worst results of the algorithms, (ii) the best results of the algorithms, (iii) the average value of the results, (iv) The *p*-value which indicates how much the algorithms’ results is produced by chance, (v) the standard deviation (STD) of the results, and (vi) the confidence interval (CI) which indicates a range in which the outcomes of the algorithms are expected to be obtained. Due to falling into the local optima solutions, the value of STD is higher than the other algorithms’ value on some datasets. For instance, the DSOS algorithm does not show stable behavior in finding a potential answer to the problem as the size of the problem increases.

**Table 2:** A comparison of the algorithms’ performance on the generated datasets

Dataset	Algorithm	Worst	Best	Average	STD	*P*-value	Low of CI	High of CI
TRA	Trader	−1.00	− 1.00	− 1.00	0.00	0	− 1.00	− 1.00
	WCC	−1.00	− 1.00	− 1.00	0.00	0	− 1.00	− 1.00
	CUK	−1.00	− 1.00	− 1.00	0.00	0	− 1.00	− 1.00
	FOA	−1.00	− 1.00	− 1.00	0.00	0	− 1.00	− 1.00
	DSOS	−1.00	− 1.00	− 1.00	0.00	0	− 1.00	− 1.00
ATE	Trader	−1.00	− 1.00	− 1.00	0.00	0	− 1.00	− 1.00
	WCC	−1.00	− 1.00	− 1.00	0.00	0	− 1.00	− 1.00
	CUK	−21.93	−14.03	− 18.40	1.98	2.00E-49	−18.96	− 17.83
	FOA	−11.72	−2.01	−6.75	3.02	7.44E-21	−7.61	−5.89
	DSOS	−11.44	−1.09	−6.20	3.37	1.61E-17	−7.16	−5.24
CAR	Trader	−1.00	−1.00	− 1.00	0.00	0	−1.00	− 1.00
	WCC	−1.99	−1.02	− 1.55	0.29	1.40E-37	−1.63	− 1.47
	CUK	−18.97	−17.00	− 17.90	0.54	1.49E-76	−18.05	− 17.74
	FOA	−6.98	− 3.15	−5.18	1.16	3.31E-34	−5.51	−4.85
	DSOS	− 9.99	−1.04	−4.90	2.63	9.86E-18	−5.65	−4.15
NIC	Trader	−2.00	−2.00	−2.00	0.00	0	− 2.00	−2.00
	WCC	−2.00	−2.00	− 2.00	0.00	0	−2.00	− 2.00
	CUK	−96.80	−61.69	−80.69	10.44	1.39E-45	−83.66	−77.73
	FOA	−45.91	−17.13	−32.25	7.71	6.68E-33	−34.44	− 30.05
	DSOS	−52.49	−2.20	−28.40	11.77	1.72E-18	−32.54	−24.26
FEL	Trader	−2.00	−2.00	− 2.00	0.00	0	−2.00	− 2.00
	WCC	−4.99	−1.06	−2.92	1.32	1.17E-20	−3.30	−2.54
	CUK	−93.19	−76.13	−83.90	5.23	5.30E-61	−85.39	− 82.42
	FOA	−51.84	−27.04	−37.57	6.79	1.25E-38	−39.50	−35.64
	DSOS	−65.78	−1.07	−31.28	8.32	1.01E-14	−37.04	−25.53
NIF	Trader	−1.00	−1.00	− 1.00	0.00	0	−1.00	− 1.00
	WCC	−5.90	−1.02	−3.42	1.48	1.60E-21	−3.84	−3.00
	CUK	− 122.51	−78.98	−99.80	11.72	1.32E-47	− 103.13	− 96.46
	FOA	−67.15	−24.92	−47.31	12.75	1.63E-30	−50.93	−43.69
	DSOS	−58.92	−2.48	−31.63	13.13	8.08E-19	−36.15	−27.11
NIS	Trader	−1.00	−1.00	− 1.00	0.58	0	−1.00	− 1.00
	WCC	−4.68	−1.01	−2.81	1.06	5.62E-24	−3.11	−2.51
	CUK	−134.77	−67.15	− 104.76	15.09	2.00E-38	− 110.19	−99.33
	FOA	−79.83	−29.24	−54.61	15.54	1.96E-29	−59.02	− 50.19
	DSOS	−91.12	−3.16	−51.46	16.01	1.02E-18	−58.86	−44.06
DOX	Trader	−109.49	−2.19	−52.34	35.19	3.66E-14	−62.35	−42.34
	WCC	−136.29	−8.11	−78.53	40.19	1.59E-18	−89.95	−67.11
	CUK	− 500.91	− 445.43	− 476.60	17.31	1.90E-72	− 481.52	− 471.68
	FOA	− 374.17	− 203.50	− 302.06	41.25	1.58E-38	−317.64	−286.47
	DSOS	− 365.21	−65.66	− 207.26	43.20	2.67E-20	− 234.47	− 180.04
PRA	Trader	−16.93	− 1.03	−8.74	4.90	5.34E-17	−10.13	−7.35
	WCC	− 160.25	−5.99	−70.80	42.14	4.89E-16	−82.78	−58.82
	CUK	−550.57	−407.07	−476.59	42.81	2.97E-53	−488.76	− 464.42
	FOA	−374.62	−236.38	− 310.26	46.38	1.48E-42	− 323.44	− 297.08
	DSOS	− 371.64	− 130.85	− 242.03	47.33	4.26E-27	− 264.08	− 219.98

*STD* Standard deviation, *CI* Confidence interval, *TRA* Trandolapril, *ATE* Atenolol, *CAR* Carteolol, *NIC* Nicardipine, *FEL* Felodipine, *NIF* Nifedipine, *NIS* Nisoldipine, *DOX* Doxazosin, *PAR* Prazosin, *WCC* World competitive contest algorithm, *CUK* Cuckoo, *FOA* Forest optimization algorithm, *DSOS* Discrete symbiotic optimization search

Every algorithm’s performance on the Trandolapril dataset is the same as the other algorithms’. Also, The best results obtained from applying both Trader and the WCC algorithm on the Atenolol and Nicardipine datasets are alike. The WCC algorithm’s best-obtained score on the Felodipine dataset is better than the other algorithms’. In the case of the newly introduced algorithm (Trader), its best-acquired outcomes are very similar to the WCC algorithm’s on the Felodipine dataset. Compared to the other algorithms, Trader has better performance on the remaining datasets.

Table 3 summarizes the data presented in Table 2 and makes a comprehensive comparison among the algorithms. This Table shows that compared to the other methods, the newly proposed method is the most suitable option for the synthetic repositioning of drugs.

**Table 3:** A comprehensive comparison of the algorithms’ performance on the generated datasets

Dataset	Algorithm	Worst	Best	Average	STD	*P*-value	Low of CI	High of CI
ALL	Trader	−15.05	−1.36	−7.79	4.45	4.07E-35	−9.05	−6.52
	WCC	−35.34	−2.47	−18.23	9.61	5.45E-37	−20.96	−15.49
	CUK	− 171.19	− 129.83	− 151.07	11.68	2.22E-39	−154.52	− 147.62
	FOA	−112.58	−60.48	−88.55	14.84	8.26E-42	−93.23	− 83.87
	DSOS	−114.18	−23.17	−67.13	16.20	1.12E-35	−75.22	−59.04

*STD* Standard deviation, *CI* Confidence interval, *WCC* World competitive contest algorithm, *CUK* Cuckoo, *FOA* Forest optimization algorithm, *DSOS* Discrete symbiotic optimization search

To make a comparison among the algorithms, their performance was examined based on the Wilcoxon statistical test [48]. For the purpose of this comparison, the results of the proposed algorithm (Trader) which were regarded as the test base, were compared with the other algorithms’ outcomes (Table 4). As mentioned earlier, the *p*-value in Table 2 shows what amount of the results of the algorithms is produced randomly, but the *p*-value in Table 4 demonstrates whether the proposed algorithm’s performance is the same as or more efficient than the others’ performance. For the purpose of comparing the proposed algorithm with an algorithm, named A, we considered the following hypotheses:

- **H**_**0**_: The performance of the proposed algorithm is the same as the performance of A.
- **H**_**1**_: The performance of the proposed algorithm is more efficient than the performance of A.

**Table 4:** The *p*-value of the algorithms on the generated datasets based on the results of the Trader algorithm (proposed algorithm) as a test base

Algorithm	TRA	ATE	CAR	NIC	FEL	NIF	NIS	DOX	PRA
WCC	1.00	1.00	9.72e-18	0.01	1.00e-05	1.17e-15	2.77e-16	3.84e-7	1.12e-13
CUK	1.00	3.00e-48	2.49e-75	4.66e-45	1.72e-60	2.14e-47	3.15e-38	4.08e-51	1.51e-52
FOA	1.00	4.50e-18	6.67e-30	1.29e-31	1.68e-37	4.33e-30	4.54e-29	3.53e-30	6.92e-42
DSOS	1.00	1.01e-14	3.99e-14	2.87e-17	9.73e-14	2.84e-18	2.19e-18	3.45e-14	1.44e-26

*TRA* Trandolapril, *ATE* Atenolol, *CAR* Carteolol, *NIC* Nicardipine, *FEL* Felodipine, *NIF* Nifedipine, *NIS* Nisoldipine, *DOX* Doxazosin, *PAR* Prazosin, *WCC* World competitive contest algorithm, *CUK* Cuckoo, *FOA* Forest optimization algorithm, *DSOS* Discrete symbiotic optimization search

If the *p*-value is less than 0.05, H_0_ will be rejected and H_1_ will be accepted. Otherwise, H_0_ will be accepted, and H_1_ will be rejected.

ii) In the second part of the results, the outcomes of the algorithm are discussed from the drug synthetic repurposing aspect. For the selected drugs against HT, Table 5 demonstrates a list of drugs which may be replaced with a given drug.

**Table 5:** The synthetic repurposing of drugs for the HT

HT drug	The selected drugs by the algorithms				
	Trader	WCC	CUK	FOA	DSOS
Trandolapril	Cilazapril	Enalapril	Fosinopril	Fosinopril	Quinapril
Atenolol	Esmolol	Esmolol	Alprenolol	Nebivolol	Practolol
Carteolol	(Xamoterol+Nebivolol)	(Xamoterol+Nebivolol)	Penbutolol	Penbutolol	Penbutolol
Nicardipine	(Cyclandelate+Nisoldipine), (Drotaverine+Nisoldipine)	(Drotaverine+Nisoldipine)	–	–	(Drotaverine+Nisoldipine)
Felodipine	(Pinaverium+ Nisoldipine)	(Pinaverium+Nilvadipine)	(Pinaverium+Mibefradil)	(Pinaverium+Nifedipine)	(Pinaverium+Nilvadipine)
Nifedipine	(Pinaverium + Nisoldipine)	–	–	–	–
Nisoldipine	(Pinaverium+Isradipine), (Pinaverium+Drotaverine+Nilvadipine)	–	–	–	–
Doxazosin	(Nicergoline)	(Droperidol)	–	–	(Dapiprazole)
Prazosin	(Dapiprazole), (Nicergoline+Tamsulosin), (Nicergoline+Periciazine+Dapiprazole)	(Alfuzosin+Flupentixol)	(Alfuzosin+Flupentixol)	(Alfuzosin+Phentolamine)	(Alfuzosin+Silodosin)

*WCC* World competitive contest algorithm, *CUK* Cuckoo, *FOA* Forest optimization algorithm, *DSOS* Discrete symbiotic optimization search

Trandolapril which is used for treating the minor HT, is a molecule which blocks the angiotensin-converting enzyme’s activity (ACE) [49]. This medication can interact with ACE whose involvement in regulating the rate of fluids in the body makes it capable of controlling HT. As shown in Table 5, all of the algorithms have proposed a drug with a few side effects, which may be replaced with Trandolapril. Atenolol, which is used to cure the abnormal rhythm of heartbeats, is a beta-blocker. Like Trandolapril, it interacts with ACE [50]. Once again, all of the algorithms have suggested a drug which can be replaced with Atenolol, as in Trandolapril’s case. However, the proposed drugs have a number of side effects and may produce some undesired effects on the body. Trader, WCC, and DSOS managed to find some substitutes without side effects and therefore are better than FOA and CUK.

Since Carteolol acts against the beta-2 adrenergic receptor and agonists the beta-1 adrenergic receptor [51] it can reduce the blood pressure; for this reason, it is regarded as a candidate for treating HT. All of the algorithms propose a list of drugs, but both their lists and their costs differ. Compared to the other algorithms, both Trader and WCC algorithm have produced a proper result based on the score function.

Nicardipine, which is a calcium channel blocker, controls the blood pressure [52]. CUK and FOA could not propose some candidate drugs which can be replaced with Nicardipine. While Trader yielded two lists which can be replaced with Nicardipine, WCC and DSOS proposed just a synthetic list instead of Nicardipine. Based on the score function, all of the lists have an identical value and may be a suitable option for treating HT.

Felodipine is capable of controlling the blood pressure by blocking the calcium channels, so it can be a proper option for curing both the mild and minor HT [53]. To treat HT, instead of proposing Felodipine, algorithms have acquired a list of medications which include Pinaverium. Trader’s suggested list has the best score value compared to the lists proposed by the other ones.

Nifedipine and Nisoldipine slow down the penetration of calcium into the heart cells and vessel walls [54]. As a result, heart can pump blood around the body and widen the blood vessels efficiently. All of the algorithms except Trader have failed to discover candidates which can be used instead of Nifedipine and Nisoldipine. In this case, Trader puts forward one or two possible lists.

Doxazosin blocks the Alpha-1A, Alpha-1B, and Alpha-1D adrenergic receptors and smooths the growth of muscle cells [55]. In contrast to CUK and FOA which did not manage to detect any drug substitutes for Doxazosin, Trader, WCC, and DSOS succeeded in discovering a single-member list. Based on the score value, Trader, WCC, and DSOS, respectively can be ranked among the most functional algorithms.

Prazosin works against the Alpha-1A, Alpha-1B, and Alpha-1D adrenergic receptors. In a comparison made among the results obtained from different algorithms, it is shown that to control HT, Trader has substituted three different lists for Prazosin, whereas the other algorithms have replaced Prazosin with just a two-member list. Moreover, from the score value viewpoint, Trader’s proposed lists are more suitable than those suggested by the other algorithms.

Based on the obtained results, it can be concluded that Trader (the newly introduced algorithm) is more efficient than the other state-of-the-art algorithms and proposes some better synthetic drug lists to treat HT. In addition, the results of the newly proposed drug lists by Trader have been presented in detail in Table 6 and demonstrate how similar the suggested drugs are.

**Table 6:** The outcomes of the proposed algorithm on the selected drugs

Given drug	Chemical formula	Proposed lists	Drugs of the list	Chemical formula	Similarity
Trandolapril	C_24_H_34_N_2_O_5_	(Cilazapril)	Cilazapril	C_22_H_31_N_3_O_5_	0.87
Atenolol	C_14_H_22_N_2_O_3_	(Esmolol)	Esmolol	C_16_H_25_NO_4_	0.75
Carteolol	C_16_H_24_N_2_O_3_	(Xamoterol+Nebivolol)	Xamoterol	C_16_H_25_N_3_O_5_	0.80
			Nebivolol	C_22_H_25_F_2_NO_4_	0.58
Nicardipine	C_26_H_29_NO_6_	(Cyclandelate+Nisoldipine), (Drotaverine+Nisoldipine)	Cyclandelate	C_17_H_24_O_3_	0.49
			Nisoldipine	C_20_H_24_N_2_O_6_	0.82
			Drotaverine	C_24_H_31_NO_4_	0.71
Felodipine	C_18_HCl_2_NO_4_	(Pinaverium+ Nisoldipine)	Pinaverium	C_26_H_41_BrNO_4_	0.35
			Nisoldipine	C_20_H_24_N_2_O_6_	0.57
Nifedipine	C_17_H_18_N_2_O_6_	(Pinaverium + Nisoldipine)	Pinaverium	C_26_H_41_BrNO_4_	0.35
			Nisoldipine	C_20_H_24_N_2_O_6_	0.90
Nisoldipine	C_20_H_24_N_2_O_6_	(Pinaverium+Isradipine), (Pinaverium+Drotaverine+Nilvadipine)	Pinaverium	C_26_H_41_BrNO_4_	0.40
			Isradipine	C_19_H_21_N_3_O_5_	0.83
			Drotaverine	C_24_H_31_NO_4_	0.69
			Nilvadipine	C_19_H_19_N_3_O_6_	0.85
Doxazosin	C_23_H_25_N_5_O_5_	(Nicergoline)	Nicergoline	C_24_H_26_BrN_3_O_3_	0.50
Prazosin	C_19_H_21_N_5_O_4_	(Dapiprazole), (Nicergoline+Tamsulosin), (Nicergoline+Periciazine+Dapiprazole)	Dapiprazole	C_19_H_27_N_5_	0.69
			Nicergoline	C_24_H_26_BrN_3_O_3_	0.47
			Tamsulosin	C_20_H_28_N_2_O_5_S	0.58
			Periciazine	C_21_H_23_N_3_OS	0.53

## Discussion

To discover the hidden applications of the existing drugs, a drug repositioning method, which is based on the newly introduced discrete optimization algorithm (Trader) and If-Then association rules, was proposed. The proposed method may reduce the toxicity of drugs and enhance their effectualness in curing diseases. To investigate the applicability of the suggested method, it was applied to the nine hypertension-related datasets, and the results were analyzed and examined from two perspectives. From the first viewpoint, it was shown that the state-of-the-art algorithms yield better outcomes than the others. The average score value and the confidence interval of the proposed algorithm (Trader) were − 7.79 and [− 9.05–6.52], respectively, which were remarkably better than those obtained from the other algorithms. Besides, while the proposed algorithm could detect some candidate lists for all of the datasets, the others failed to do so. For example, the other algorithms except for Trader failed to explore for the potential drugs which can be replaced with Nifedipine and Nisoldipine. From the second viewpoint, the application of the suggested drugs was examined in treating hypertension. A detailed examination of the data yielded some important results as follows: First, the proposed method succeeded in suggesting some proper substitutes for the selected drugs, which may be more useful than the current therapeutic methods in treating HT. For example, the proposed approach managed to replace Atenolol with Esmolol which belongs to a family of beta-blocker drugs and does not have any side effects [56]. As mentioned before, Atenolol can interact with two targets [57], one of which is responsible for HT, and the next one is a side effect. The small doses of the above-mentioned drugs may combine with their substitutes and produce the desired effect. For instance, in databases, it has been reported that a combination of Trandolapril and Cilazapril might prove more useful in controlling HT [58]. Second, the proposed approach can introduce the novel applications of some drugs. For example, Pinaverium which is a first-line option for curing bowl dysfunctionality, operates as an inhibitor and antagonist of the voltage-dependent calcium channel protein [59]. Moreover, more than 60 countries are exploiting Pinaverium to treat gastrointestinal disorders. However, it still has not won approval from FDA. Pinaverium’s application in controlling HT may be based on the kind of method employed in this research. Therefore, the introduced approach might especially revitalize the projects which have already failed due to the various undesired biological effects of drugs [60]. Besides, this method might be a suitable therapeutic plan for treating orphans or diseases because developing an efficient drug is both costly and time-consuming and therefore not affordable for drug companies [61]. Another introduced medication for managing HT is Dapiprazole which is an alpha-blocker agent. Dapiprazol helps to reduce the size of the pupils of the eyes in patients who suffer from mydriasis [62]. Although different studies have reported many undesired side effects of Dapiprazole and some other drugs such as Pinaverium [63], the proposed approach aims to employ these drugs in an appropriate manner. To this end, such medications are used in small doses to decrease their undesired effects, [64]. Furthermore, a proper combination of drugs might produce a synergistic effect on treating HT [65]. A few studies have investigated the synergic effects of drugs and for this purpose, have examined some drug-drug adverse reactions. However, most of them have not been specified, yet. The machine learning approaches can help predict the drug-drug adverse reactions and introduce the most reliable synthetic candidates.

## Conclusion

A novel discrete algorithm, named Trader, was introduced for the synthetic repurposing of drugs. This method can resume most of the failed drug discovery projects and might be the most suitable option for treating the orphan and rare diseases. The proposed approach takes account of the various aspects of the synthetic repositioning of medications, including the drugs’ mechanism of action on targets, the drug-drug adverse reactions, and the total number of side effects. Based on the obtained results, it can be concluded that the-state-of-the-art algorithms yield better results and show more suitable performance in comparison to the others. Furthermore, the literature findings validate the functionality of the proposed method and suggest several synthetic repurposing lists to reduce hypertension.

## Methods

**Fig. 3:**
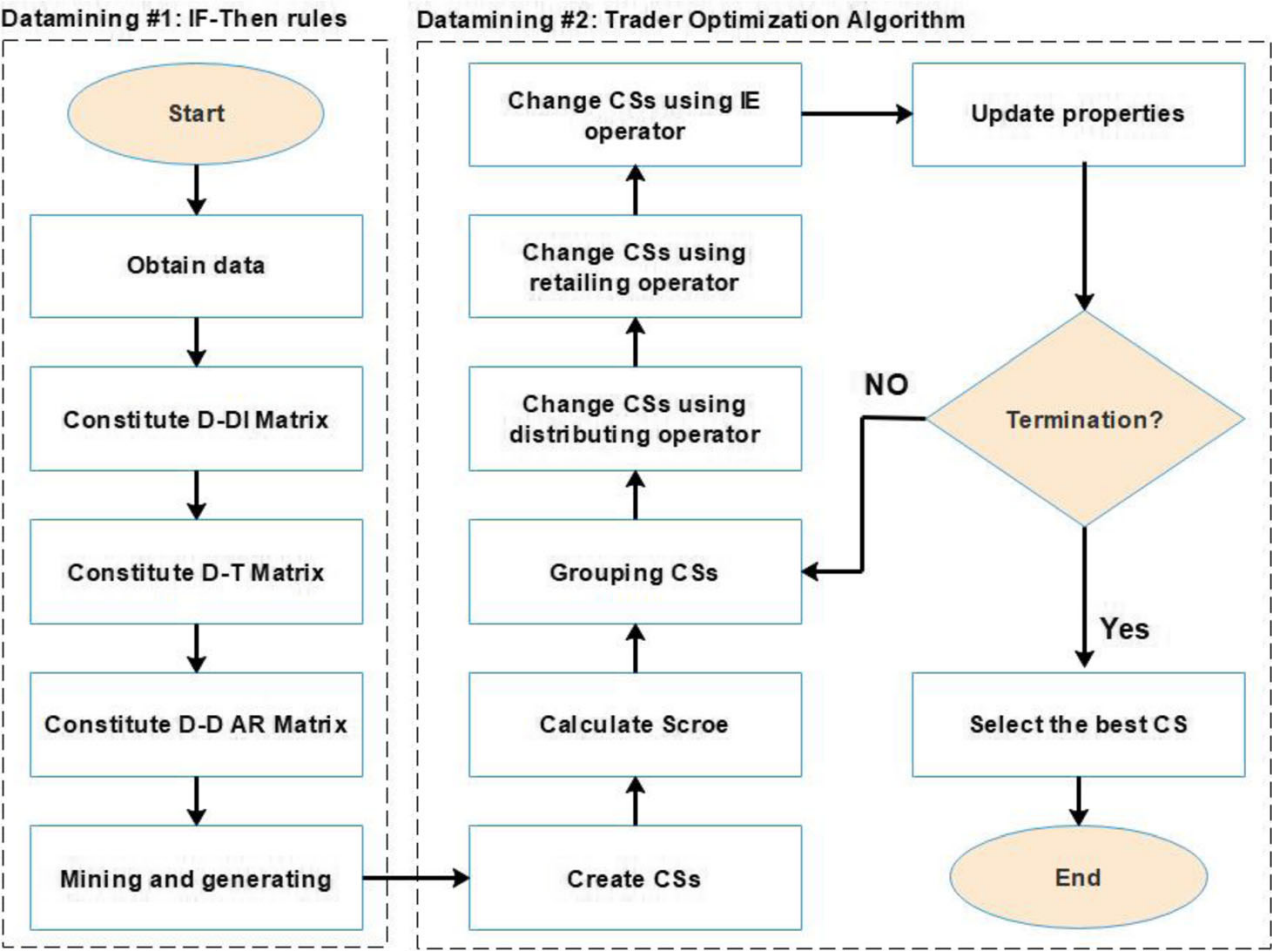
The framework of the proposed method. In the first step, the desired data are obtained from DrugR+ database. Next, drug-disease (D-DI), drug-target (D-T), and drug-drug-adverse reaction (D-D AR) matrices are formed. Drugs which can affect the HT inducing targets, are acquired based on the If-then rules. In the second step, the proposed optimization algorithm (Trader) is used to select a combination of drugs for the repurposing of medications for HT

A two-step datamining method was proposed to discover drugs that might be useful in treating HT. In the first step, based on the If-Then rules, it was determined which drugs may inhibit or prevent the targets inducing HT. In the second step, an optimal subset of the candidate drugs whose combinations may be helpful to treat HT, was selected by the proposed discrete optimization algorithm (Trader). Figure 3 presents the framework of the proposed method.

The first part of the proposed approach includes a number of steps which are as follows:

i) Obtaining the data: data on the drugs and their different targets were extracted from DrugR+ (version 2019.11) [66], which is a relational database and integrates DrugBank (version 5.0) [67] and KEGG (version *September 1, 2019*) [68] databases. DrugR+ database provides an online tool that takes a drug and suggests some potential drugs which can be substituted for the first one [69]. Meanwhile, DrugR+ has an advanced search section in which users can state their SQL queries and download the results immediately. In selecting the datasets, the size of the data and the various treatment properties of drugs have been considered.
ii) Constituting the drug-disease (D-DI) matrix: based on the downloaded data, a binary matrix, named DDI, and incorporated drugs-diseases relationships, was formed using Eq. (1).

1 $$ \mathrm{DDI}\left(\mathrm{i},\mathrm{j}\right)=\left\{\begin{array}{c}1\kern0.5em if\ the\ {i}^{th}\  drug\ is\ used\ for\ treating\ the\ {j}^{th}\  disease\\ {}0\kern0.5em else\kern23.5em \end{array}\right. $$

Where i and j indicate a drug and a disease, respectively. The total number of drugs and diseases are 13,251 and 3318, respectively.

iii) Constituting the drug-target (D-T) matrix: this matrix shows whether a drug affects a target or not. The extracted data show that there are four classes of drug-target effects, including (a) agonizing, (b) antagonizing, (c) inhibiting, and (d) inducing. The targets whose total numbers were 4893, consisted of both proteins and enzymes. The DT matrix was formed using Eq. (2).

2 $$ \mathrm{DT}\left(\mathrm{i},\mathrm{j}\right)=\left\{\begin{array}{c} INH\kern2em if\ the\ {i}^{th}\  drug\ inhibits\ the\ {j}^{th}\  target\kern2.5em \\ {} IND\kern2em if\ the\ {i}^{th}\  drug\ induces\ the\ {j}^{th}\  target\kern2.5em \\ {} AGO\kern2em if\ the\ {i}^{th}\  drug\ agonists\ the\ {j}^{th}\  target\kern2em \\ {} ANT\kern2em if\ the\ {i}^{th}\  drug\ antagonists\ the\ {j}^{th}\  target\kern0.5em \\ {}0\kern2.5em else\kern18em \end{array}\right. $$

iv) Constituting the drug-drug adverse reaction (DDAR) matrix: Some drugs may interact, neutralize their effects, and trigger a serious problem in a body. A matrix, named DDAR, was formed to investigate the problem (Eq. (3).

3 $$ \mathrm{DDAR}\left(\mathrm{i},\mathrm{j}\right)=\left\{\begin{array}{c}1\kern0.5em if\ the\ {i}^{th}\  drug\  has\  adverse\ reaction\ with\ the\ {j}^{th}\  drug\\ {}0\kern0.5em else\kern25.25em \end{array}\right. $$

v) Mining: during every stage of the process, we employed the DT and DDI matrices to extract some information which were used as the input of the discrete optimization algorithm (Trader). In the first part of the proposed method, different targets of blood pressure along with their effects (inhibiting, inducing, agonizing, and antagonizing) are determined and placed in a set named HT_TARGETS. Equation (4) presents the mentioned set as follow:

4 $$ \mathrm{HT}\_\mathrm{TARGETS}=\left[\left({\mathrm{T}}_1,{\mathrm{R}}_1\right),\dots, \left({\mathrm{T}}_{\mathrm{n}},{\mathrm{R}}_{\mathrm{n}}\right)\right] $$

Where n, T_i_, and R_i_ are the total number of the obtained targets, the i^th^ target, and the effect which leads to high blood pressure, respectively.

Furthermore, another set, named DRUGS, was created for drugs that directly interact with the HT_TARGETS collection and exert exactly the same impact on the targets. For instance, both the *Angiotensin II* and *Candesartan* drugs interact with the *Type-1 angiotensin II receptor*. However, *Angiotensin II* affects the mentioned target as an agonist whereas *Candesartan* affects it as an antagonist. Therefore, *Angiotensin II* is ignored because its function is not exactly the same as *Candesartan’s*.

In this study, the interaction between a drug such as D and a target such as T (protein (P) and an enzyme (E)) is presented by “➔”. The effect of D on T and the cause of hypertension due to T are shown by F(D,T) and F(HT,T), respectively. Next, the following rules are applied to all of the existing drugs, and the candidate drugs are added to the DRUGS set.

- IF D ➔ T && F(D,T) = F(HT,T) THEN
  D may be useful for controlling HT
- IF E ➔ P && F(E,P) = F(HT,P) THEN
  The drug, which interacts with E, may be useful for controlling HT
- IF P ➔ E && F(E,P) = F(HT,E) THEN
  The drug, which interacts with P, may be useful for controlling HT
- IF P_1_ ➔ P_2_ && F(P_1_,P_2_) = F(HT,P_2_) THEN
  The drug, which interacts with P_1_, may be useful for controlling HT
- IF E_1_ ➔ E_2_ && F(E_1_,E_2_) = F(HT,E_2_) THEN
  The drug, which interacts with E_1_, may be useful for controlling HT.

In the second part of the proposed method, the discrete optimization algorithm (Trader) was applied to select the optimal subsets of the obtained drugs which may reduce the pressure of blood. For this purpose, a number of steps were followed:

i) Creating the first population of the candidate solutions (CSs): Trader begins with randomly created potential answers, which are presented by an array shown in Eq. (5).

5 $$ \mathrm{CS}=\left[{\mathrm{V}}_1,{\mathrm{V}}_2,\dots, {\mathrm{V}}_{\mathrm{m}},\mathrm{G},\mathrm{Score}\right] $$

Where V_i_, m, G, and Score are the i^th^ variable, the total number of variables, the group, and the score or fitness of the CS. Every variable shows a drug whose value is set 1 or 0 for the selected and unselected drugs, respectively.

ii) Calculating the score of the CSs: The CSs which are answers to the mentioned problem, differ widely in terms of how much they are worthy of consideration. In this study, the score is calculated using Eq. (6).

6 $$ \mathrm{Score}= AT-\frac{\sum_{i=}^m{SE}_i}{\sum_{i=1}^m{v}_i} $$

Where m, v_i_, SE, and AT represent the length of a CS, the value of the i^th^ variable (0 or 1), the total number of side effects related to the i^th^ drug, and the total number of the covered targets which are correspondent with HT, respectively.

iii) Grouping CSs: The total number of the groups and the total number of the traders are the same concepts, and they show a group. At the beginning of the algorithm, the total number of the members in the groups is the same, and In the next iterations, they are updated using Eq. (7).

7 $$ {\mathrm{TM}}_{\mathrm{i}}=\mathrm{round}\left(\ \frac{property_i}{\sum_{k=1}^T{property}_k}\times \left(\mathrm{C}-\mathrm{M}\times \mathrm{T}\right)\right) $$

Where TM_i_, C, and T present the total number of the members in the i^th^ group, the total number of CSs, and the total number of the traders or groups, respectively. M is a constant value (2) and guarantees that none of the groups will be eliminated during the iterations of the algorithm in each stage. The property of the i^th^ trader is calculated using Eq. (8).

8 $$ {\mathrm{property}}_{\mathrm{i}}={\sum}_{i=1}^M score\left({CS}_i\right) $$

Where M and *score* show the total number of CSs in the i^th^ group and the score of the related CS, respectively. In other words, the property of a group is equivalent to the sum of its CSs’ scores.

iv) Changing the CSs: there are three operators who change both the master and the slave CSs. As shown in Eqs. (9, 10, and 11), these operators, named retailing, distributing, and importing-exporting, try to improve the CSs. when the retailing operator is employed, the minimum number of changes will be applied to a slave-CS. The distributing operator obtains some values from the best CS of the group (the master-CS) and then, assigns them to the other CSs of the group (slave_CS). While both the distributing and retailing operators change the slave-CSs, the importing-exporting operator brings about changes in the master-CSs. The changes can be accepted in all of the operators, provided that they improve the value of a CS’s score.

9 $$ {\sum}_{i=1}^R\left({CS}_{slave}(K)=\left|\left({CS}_{slave}(K)-1\right)\right|\right) $$

Where K and R are two random integer values in [1, length(CS)] and [1, length(CS)/10], respectively.

10 $$ {\sum}_{\mathrm{i}=1}^R\left({CS}_{slave}(K)={CS}_{master}(K)\right) $$

Where K and R are two random integer values in [1, length(CS)].

11 $$ {\sum}_{i=1}^R\left({CS_{master}}_j(K)={CS_{master}}_m(K)\right) $$

Where j and m are the importer and exporter CSs, respectively. Also, the values of K and R can be measured using Eq. (9).

**Fig. 4:**
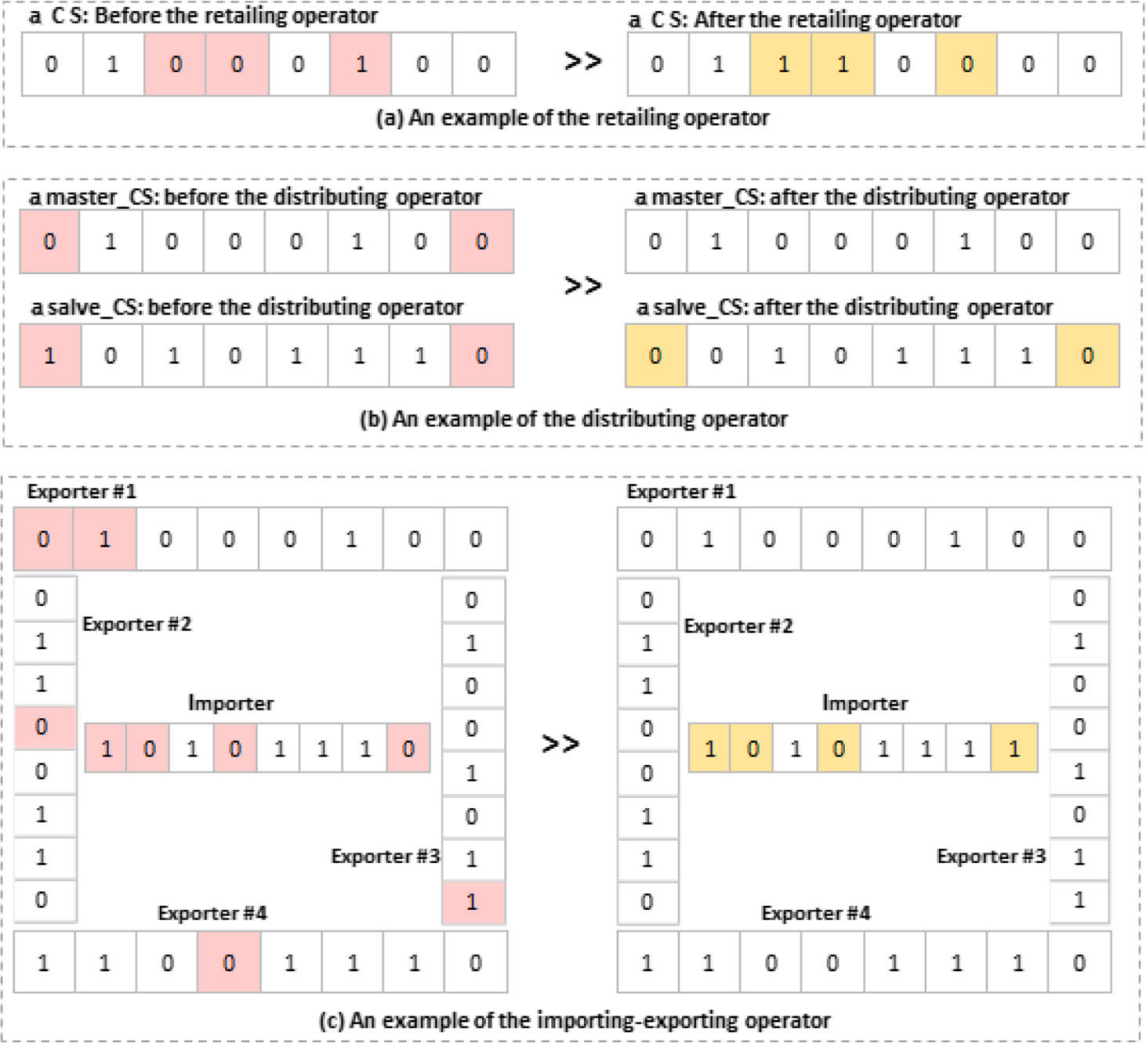
The Trader’s operators. **a** An example of the retailing operator: three points have been chosen randomly, and, then, their values have been updated by Eq. (9). This operator is only applied to slave_CSs. **b** An instance of the distributing operator: The master_CS selects two values from its values and distributes them to the slave_CS. **c** An example of the importing-exporting operator: The exporter CSs select some of their values and send them to the importer CS. After applying the operators, the score function is called. The changes can be accepted, provided that the new score is better than the previous one. In contrast, the changes will be ignored, if the previous values are retrieved from the memory

An instance of the mentioned operators has been illustrated in Fig. 4.

### Calculating drug-drug similarity score

Equation (12) was employed to calculate the similarity score between drugs [70]:

12 $$ \mathrm{Similarity}\Big({\mathrm{D}}_{\mathrm{i}},{\mathrm{D}}_{\mathrm{j}}=\frac{\sum_{r=1}^n\left({w}_{r\times }{C}_{i.r}\times {C}_{j.r}\right)}{\sum_{r=1}^n\left(\sqrt{w_{r\times }{C}_{i.r}}\sqrt{w_{r\times }{C}_{j.r}}\right)} $$

Where D_i_, D_j_, n, C_i,r_, and C_j,r_ are the i^th^ drug, the j^th^ drug, the total number of the chemical components, the total number of the r^th^ chemical component in i^th^, and the total number of the r^th^ chemical component in the j^th^ chemical component, respectively. W_r_, which is calculated by Eq. (13), is the weight of the r^th^ chemical component.

13 $$ {\mathrm{w}}_{\mathrm{r}}=\frac{\min \left({d}_r.\kern0.5em 1\right)\times \min \left({C}_{i.r}.\kern0.75em {C}_{j.r}\right)}{\max \left({C}_{i.r}.\kern0.5em {C}_{j.r}\right)+ eps} $$

Where d_r_ shows the data frequency of the r^th^ chemical component.

## Data Availability

All the source codes are available in the following link: https://github.com/LBBSoft/Trader. In addition, data are accessible using our developed database DrugR+ (http://drugr.ir/).

## Abbreviations

ACE: Angiotensin-converting enzyme
ATE: Atenolol
CAR: Carteolol
CI: Confidence interval
CS: Candidate solution
CUK: Cuckoo optimization
D-DI: Drug-disease
D-T: Drug-target
DDR: Drug-drug adverse reaction
DOX: Doxazosin
DSOS: Discrete symbiotic optimization search
FEL: Felodipine
FOA: Forest optimization algorithm
HT: Hypertension
NIC: Nicardipine
NIF: Nifedipine
NIS: Nisoldipine
PAR: Prazosin
STD: Standard deviation
TNC: Total number of candidates
TNMT: Total number of main targets
TNT: Total number of targets
TRA: Trandolapril
WCC: World competitive contests
